# A Structural and Functional Perspective of Death Receptor 6

**DOI:** 10.3389/fphar.2022.836614

**Published:** 2022-03-24

**Authors:** Xiuying Ren, Zhi Lin, Wensu Yuan

**Affiliations:** School of Life Sciences, Tianjin University, Tianjin, China

**Keywords:** death receptor, DR6, death domain, signaling, co-receptor

## Abstract

As a member of the tumor necrosis factor receptor superfamily (TNFRSF), death receptor 6 (DR6) has a similar structural architecture to other family members. The extracellular region of DR6 contains four cysteine-rich domains, followed by a single-pass transmembrane domain and an intracellular region. Since its discovery, DR6 has become an orphan receptor ubiquitously expressed to transduce unique signaling pathways. Although the free ectodomains of β-amyloid precursor protein (APP) can bind to DR6 to induce apoptotic signals, the natural ligands of DR6 still remain largely unknown. In this review, we focus on recent research progress of structural and functional studies on DR6 for better understanding DR6-mediated signaling and the treatment of DR6-related diseases.

## Introduction

Tumor necrosis factor (TNF) family members and their receptors have been recognized as key players in mediating the growth, differentiation, apoptosis, and survival of normal cells. Abnormal expression, activation, or functional changes of these family members can lead to various diseases, including rheumatoid arthritis, inflammatory bowel disease, Alzheimer’s disease (AD), type 2 diabetes, and cancer (Uysal et al., 1997; Bolger and Anker, 2000; Lans et al., 2001; Hayashi and Faustman, 2003). Tumor necrosis factor receptor superfamily (TNFRSF) has 27 members, such as TNFR1 (tumor necrosis factor receptor 1, also known as TNFRSF1A), p75^NTR^ (p75 neurotrophin receptor, TNFRSF16), death receptor 3 (DR3, TNFRSF25), DR4/5 (TNFRSF10), DR6 (TNFRSF 21), et al. (Haase et al., 2008). The signal transduction pathways activated by the interaction between the receptors and ligands are the key to revealing the physiological functions of the receptors (Anderson et al., 1997).

DR6 was first discovered and identified as a member of TNFRSF more than 20 years ago (Pan et al., 1998). Human DR6 gene, which is located in chromosome No. 6 (6p21.1–21.1), is ∼78 kb long and transcribed into a 655 amino acid chain with a theoretical molecular weight (MW) of ∼71.8 kDa. Its mRNA is widely expressed in heart, brain, placenta, pancreas, and immune organs such as thymus, spleen, lymph nodes, and bone marrow. The expression of DR6 in bladder and brain tissue is particularly abundant, while it is relatively low in adult liver and peripheral blood cells (Pan et al., 1998). Compared with normal tissues, DR6 is also expressed at high levels in many tumors cell lines, such as colorectal cancer, lung cancer, melanoma, breast cancer, and prostate tumor (Pan et al., 1998). Following the discovery, very limited progress was made in the identification of extracellular ligands and intracellular partners of DR6, and our understanding of molecular and structural mechanisms underlying DR6 signaling is still poor. In this review, we focus on recent advances in the structures and functions of DR6 for better understanding DR6-mediated signaling and the treatment of DR6-related diseases.

## Structure of DR6

### Domain Structures of DR6

**FIGURE 1:**
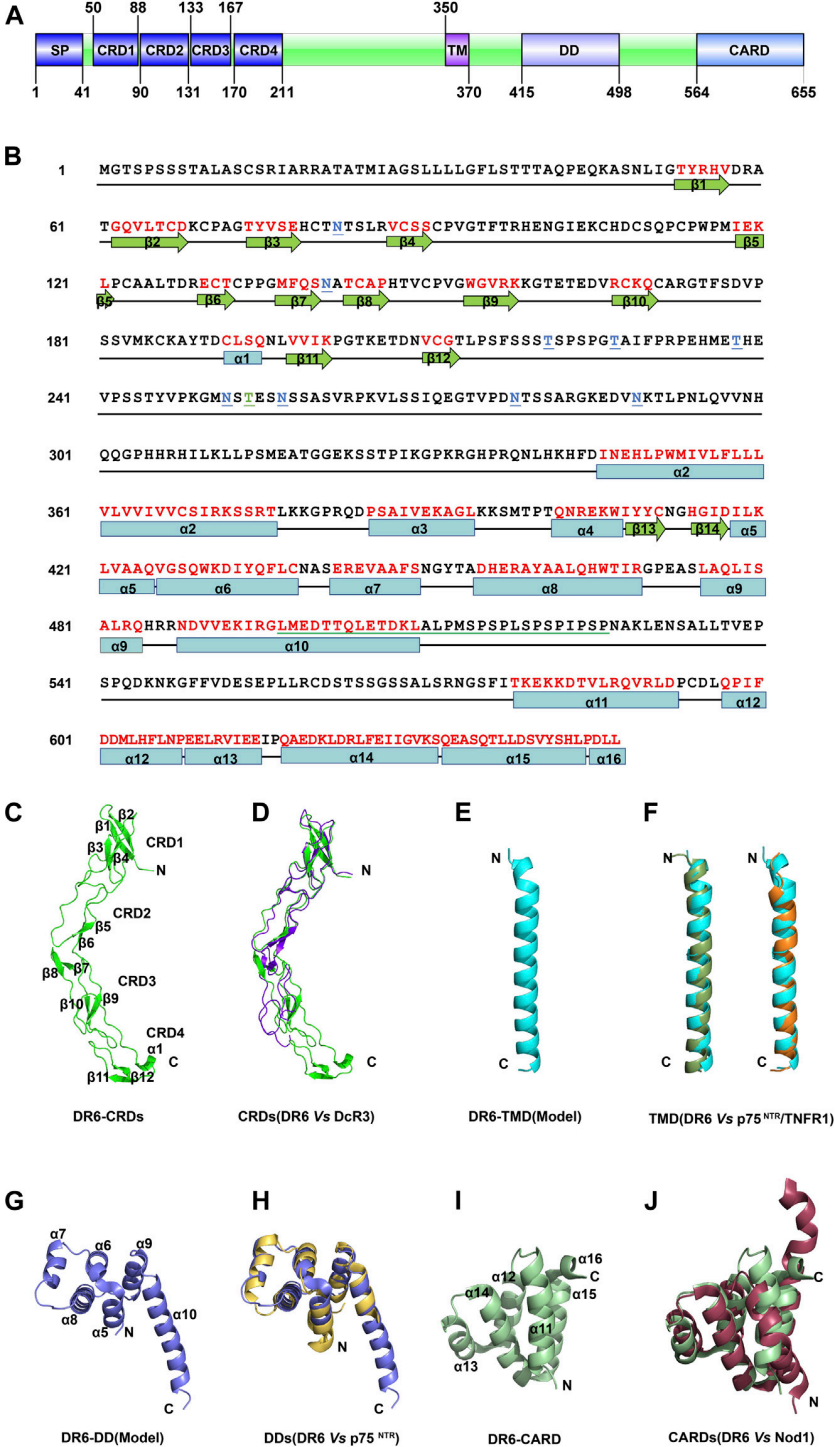
The structure of DR6 **(A)** Molecular architecture of human DR6. SP: signal peptide; CRD: cysteine-rich domain; TM: transmembrane sequence; DD: death domain; CARD: caspase activation and recruitment domain. **(B)** Amino acid sequence of full-length human DR6. The secondary structures of DR6 are shown below the sequence. The red letters indicate the structured elements. N-glycosylation sites are colored in blue and underlined; O-glycosylation sites, green and underlined. A putative leucine zipper motif with a proline-rich sequence is green underlined **(C)** Ribbon drawing of crystal structure of the DR6-CRDs (PDB ID: 3QO4) **(D)** Structural comparison between CRDs of DR6 (green) and DcR3 (purple, PDB ID: 3MHD) **(E)** Structural model of the DR6-TMD. **(F)** Structural comparison between monomeric TMDs of DR6 (cyan) and p75^NTR^ (dark green, left, PDB ID: 2MIC)/TNFR1(orange, right, PDB ID: 7K7A) **(G)** Structural model of the DR6-DD. **(H)** Structural comparison between DDs of DR6 (blue) and p75^NTR^ (yellow, PDB ID: 4F42) **(I)** Ribbon drawing of the lowest energy structure of C-terminal CARD domain of DR6 (PDB ID: 2DBH) **(J)** Structural comparison between CARDs of DR6 (green) and Nod1 (dark red, PDB ID: 2NZ7).

DR6 is a type I transmembrane death receptor. A predicted signal peptide was identified from residues Met1 to Ala41 (Figure 1 A and B). The extracellular region of DR6 contains four cysteine-rich domains (CRDs). The transmembrane domain (TMD) is composed of ∼21 amino acids (AAs, 350-370). A putative death domain (DD, 415-498) and an unidentified caspase activation and recruitment domain (CARD)-like domain (564-655) are located in the cytoplasmic region. Between DD and CARD-like domain, a putative leucine zipper motif with a proline-rich sequence was also identified with unknown function. It is evident that the structure of the intracellular region of DR6 is the most unique in the death receptor family. DR6 is also a highly glycosylated protein. All six extracellular asparagines in the extracellular region (Asn82, Asn141, Asn252, Asn257, Asn278, and Asn289) are N-glycosylation sites, and a cluster of serines and/or threonines between residues 212 and 254 juxtaposed to the CRDs are potential sites for mucin-type O-glycosylation. Interestingly, an *S*-palmitic acid modification site at Cys368 in the predicted transmembrane domain was also found (Klíma et al., 2009). However, this palmitoylation is not required for directing DR6 into lipid rafts.

The 3D structure of CRDs of DR6 (42–218) was determined as a monomer by X-ray crystallography (Kuester et al., 2011). As shown in Figure 1C, its secondary structure includes six antiparallel β sheets and one short α helix. However, the degree of structure is very low, and nearly two-thirds of residues in CRDs do not fold into any standard secondary structures. The overall structures of CRDs are similar to those of other death receptors such as decoy receptor 3 (DcR3) (Zhan et al., 2011), although their sequence identities are not high (<50%) (Figure 1D). SAXS analysis revealed that the intact ectodomain (1–348) of DR6 exists as a dimeric form in solution although its high-resolution structure has not been determined (Ru et al., 2012). The homo-dimerization site of DR6 ectodomain is likely located in the residues between 219 and 348, which doesn’t include CRDs. This may explain why the DR6-CRDs protein was crystallized as monomers while the whole ectodomain is a dimer in solution. Since the dimerization site is very close to the membrane, it is possible that the DR6-TMD may also dimerize or oligomerize in the membrane. The DR6-TMD has a conserved proline-containing motif which also exists in many members of TNFRSF (Zhao et al., 2020). However, sequence determinants for TMD oligomerization are vastly diverse, and how the DR6-TMDs homo-associate in the membrane remains largely unknown due to unavailability of the structure of the DR6-TMD. Structural modeling of the DR6-TMD monomer by AlphaFold suggests a single-pass transmembrane domain with an ⍺-helical conformation (Figure 1E), which is common in other known TMDs of TNFRSF members, including p75^NTR^, Fas, TNFR1, and DR5 (Fu et al., 2016; Nadezhdin et al., 2016; Goh et al., 2018; Fu et al., 2019; Zhao et al., 2020; Jumper et al., 2021). Structural comparison shows that the predicted DR6-TMD is more similar to TMDs of p75^NTR^ and TNFR1 with root mean squared deviations (RMSDs) of ∼1.5–1.6 Å in the structural regions despite their low sequence identity of <20% (Figure 1F). Although the DR6-TMD also has a cysteine residue (Cys368), it is unclear if the DR6-TMD could form a disulfide-bonded dimer in the membrane since Cys368 was shown to be *S*-palmitoylated (Klíma et al., 2009).

The putative DD of DR6 is predicted to consist of six helices. Its model structure shows high similarity to other DDs, especially the p75^NTR^-DD with an RMSD of ∼1.6 Å, although the length of the last helix could be different (Figure 1 G and H) (Lin et al., 2015; Yuan et al., 2019). This strongly suggests that DR6 may harbor a canonical DD in the middle of its intracellular region. Solution structure of the C-terminal domain of DR6 also shows that it folds into a six-helix bundle (Figure 1I). Differently, the last helix of this domain is only composed of three amino acid residues (DLL) which have hydrophobic interactions with helices α11 and α15. It appears that this short helix is important to stabilize the protein global folding. The overall topology of the C-terminal domain of DR6 is very similar to those of other CARD domains, such as nucleotide-binding oligomerization domain-containing protein 1 (NOD1)-CARD (Figure 1J), with DALI (distance matrix alignment) Z-scores of >8.0 (Holm, 2019). Therefore, this C-terminal domain may be classified into CARD subdomain in the DD superfamily. Since the linker between the putative DD and the CARD domain is unusually long (>60 AA residues), it is not clear if the DR6-DD could directly interact with the DR6-CARD for the recruitment of downstream adaptor proteins. The structural mechanism of DD and CARD-mediated intracellular signaling upon receptor activation remains completely unknown.

### Complex Structure Between DR6 and APP

**FIGURE 2:**
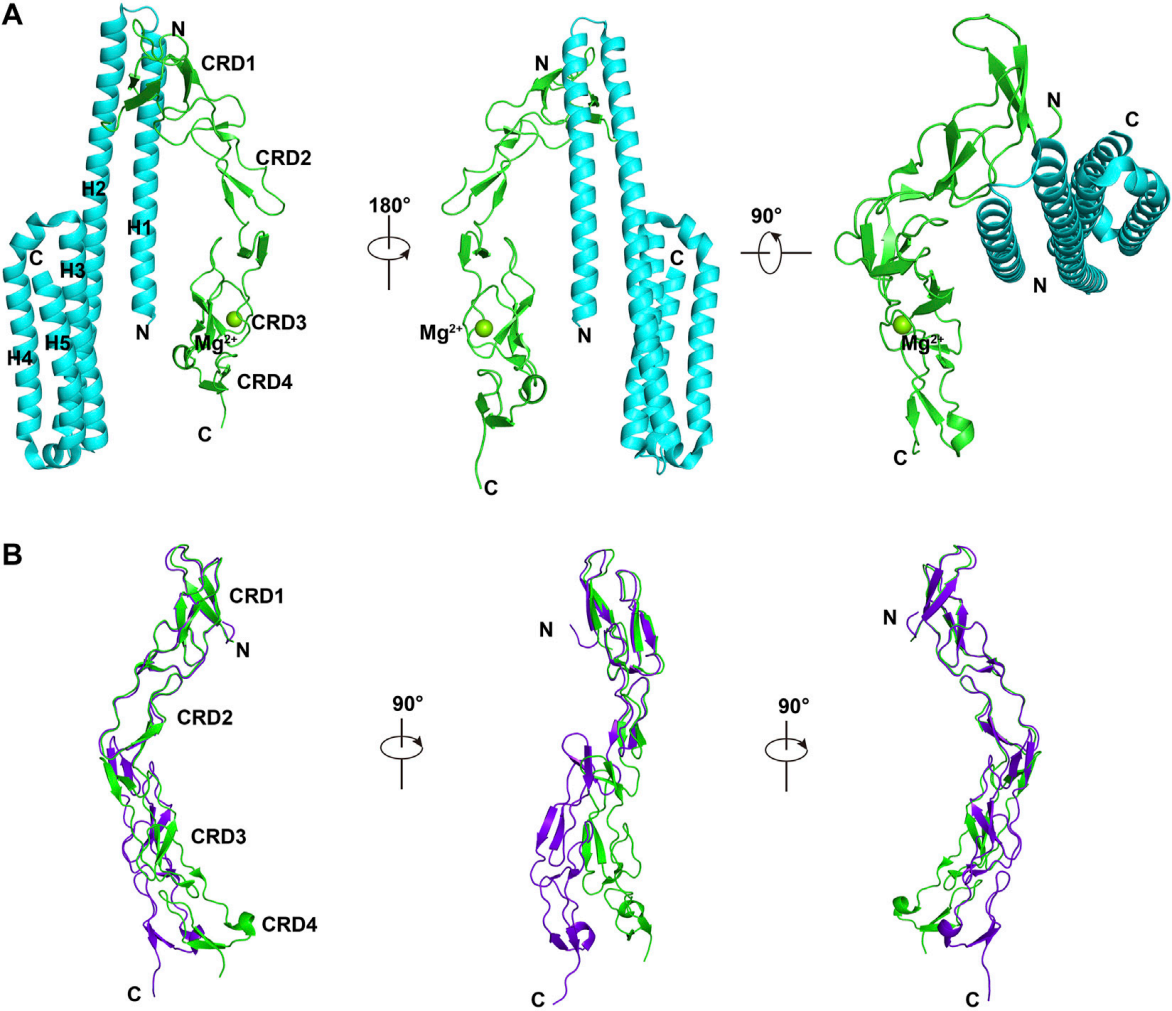
Complex structure between DR6-CRDs and APP-E2 (PDB ID:4YN0) **(A)**. APP-E2 domain is colored in cyan, and CRDs of DR6 are colored in green. **(B)**. Structural comparison between monomeric DR6-CRDs (purple) and the DR6-CRDs (green) from DR6-CRDs:APP-E2 complex.

The ectodomain of DR6 has been shown to bind to the ectodomain of β-amyloid precursor protein (APP) to stimulate axon pruning and inhibit synapse formation (Xu et al., 2015). APP is a member of type I transmembrane proteins. It consists of a short cytoplasmic C-terminal tail and a large extracellular region including E1 and E2 domains connected by an acidic non-structural sequence (Coburger et al., 2014). The complex structure between the E2 domain of APP and CRDs of DR6 (mouse) shows two small interfaces (Figure 2A). The major interaction occurs between helices H1 and H2 of APP-E2 and CRD1 of DR6. Both hydrophobic and electrostatic interactions, including strong salt bridges, are identified in the binding interface. Due to the flexible linker between CRD2 and CRD3, binding of CRDs of DR6 to the APP-E2 domain leads to significant conformational changes in CRDs (Figure 2B). This domain re-arrangement results in the formation of the minor interface between CRD3 of DR6 and helix H1 of APP-E2 (Figure 2A) (Kuester et al., 2011). Since the complex structure between dimeric ectodomains of DR6 and APP is unavailable, the precise mechanism of DR6 activation upon APP binding is still not fully understood.

## DR6 Signaling Pathway

Most death receptors mediate signal transduction through the intracellular DD-containing adaptor protein, such as Fas-associated death domain (FADD) and tumor necrosis factor (TNF) receptor type 1-associated death domain protein (TRADD) (Lavrik et al., 2005; Wilson et al., 2009; Zhang et al., 2017; Li et al., 2020; Ning Zhang et al., 2021). Different adaptor proteins can also interact with each other to propagate specific signals. Early studies reported that DR6 can only weakly interact with TRADD but it failed to bind most of known adaptor proteins, including FADD, receptor interacting protein (RIP), and RIP-related ICH-1/CED-3 homologous protein (RAIDD) (Smith et al., 1994; Pan et al., 1998; Hu et al., 2014). In addition, DR6-mediated apoptosis is cell-dependent. For example, DR6 expression could significantly induce the apoptotic pathway in prostate cancer cell line LnCAP, but no obvious apoptosis was observed in other prostate cancer cell lines, such as PC3 and DU145 (Kasof et al., 2001). It is likely that the cellular signaling pathways mediated by DR6 could be very different from other death receptors.

### Activation of DR6

**FIGURE 3:**
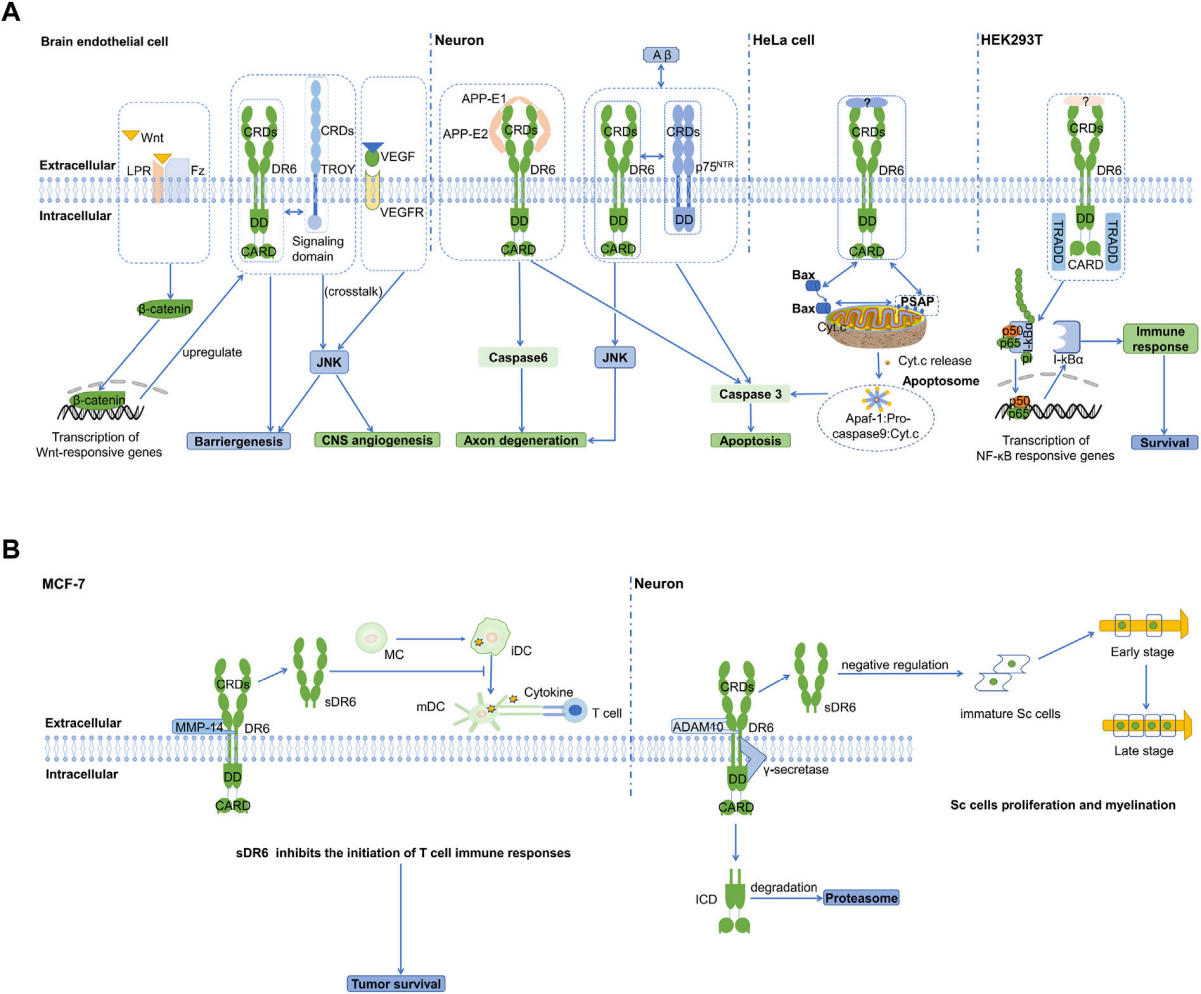
Cartoon models of DR6 signaling **(A)** DR6-mediated intracellular signaling pathways are showed in four different cells, brain endothelial cell, neurons, HeLa, and HEK293T. In brain endothelial cell, DR6 and TROY complex is involved in angiogenesis and BBB formation. They are regulated by Wnt/β-catenin signaling and engage in crosstalk with the VEGF/VEGFR2 signaling pathway. In neuron cells, DR6 can interact with APP or p75^NTR^ to promote neuronal apoptosis and axonal degeneration. In HeLa cells, DR6 can promote mitochondria-dependent apoptosis through membrane protein PSAP. In HEK293T, DR6 can induce NF-kB pathway and promote cell survival through adaptor protein TRADD. **(B)** Extracellular signaling pathway of sDR6. In MCF-7 cancer cell line, cleavage of the extracellular region of DR6 by the metalloenzyme MMP-14 leads to the formation of sDR6. This soluble protein can hinder the differentiation of monocytes into immature dendritic cells, thereby inhibiting the body’s immune response. In neurons, sDR6 produced by metalloprotease ADAM10 cleavage can trans-act on Schwann cells and inhibit their proliferation and myelination in the PNS. The intracellular domain is released into the cytosol and rapidly degraded by proteasome.

Currently, the only identified ligand for DR6 is the extracellular fragment of APP. In 2009, Nikolaev et al. found that DR6 is involved in the death process of normal neuron cell bodies (Nikolaev et al., 2009). It also participates in the pruning process of neurons. Interaction between ectodomains of APP and DR6 can signal apoptosis of neuronal cell bodies through caspase 3 and axon degeneration through caspase 6 *in vitro* and *in vivo* (Figure 3A) (Nikolaev et al., 2009). The amino-terminal fragment of APP (N-APP, or E1 domain of APP) was found to be cleaved from the membrane after trophic-factor deprivation and then interact with DR6 to trigger degeneration. Therefore, APP and DR6 were proposed as important components involved in a neuronal self-destruction pathway observed in Alzheimer’s disease. How the extracellular signals are transduced to activate the downstream caspases in the neuron cells remains largely unknown.

### DR6 Co-receptors

Besides the transmembrane protein APP, DR6 can interact with other receptors to transduce signals together. TROY (TNFRSF expressed on embryo) was shown to physically interact with DR6 through their transmembrane or cytoplasmic domains and form co-receptors during CNS vascular development (Figure 3A) (Tam et al., 2012). This functional death receptor complex is involved in CNS-specific angiogenesis and blood-brain barrier (BBB) formation, mainly via regulation of sprouting activity. Further investigation found that they engage in signaling crosstalk with vascular endothelial growth factor (VEGF)-mediated nonapoptotic JNK activation and brain endothelial sprouting. In addition, DR6 and TROY also genetically interact with each other and are downstream target genes of canonical Wingless-type protein (Wnt)/beta-catenin signaling. They are transcriptionally co-regulated at the BBB through neuroepithelium-derived Wnt ligands and exhibit overlapping expression patterns. The ligand for initiating DR6 and TROY signaling has not been identified. However, we cannot rule out the possibility they may function in a ligand-independent manner.

Another important TNFRSF member, p75^NTR^, has also been shown to form a complex with DR6 to mediate Aβ-induced cortical neuron death through caspase 3 in the absence of neurotrophin (NT) (Figure 3A) (Hu et al., 2013). Blocking of the formation of DR6:p75^NTR^ receptor complex can significantly reduce Aβ-induced neurotoxicity and cell death. The cytoplasmic DD of DR6 is essential for the co-receptors to activate caspase 3. Nevertheless, the structural mechanism of the interaction between these two receptors is completely unknown. Very recently, it was reported that p75^NTR^ can interact with APP at the plasma membrane and regulate APP internalization and amyloidogenic processing (Yi et al., 2021). Although p75^NTR^ has a lower affinity for APP than DR6, it is tempting to speculate that p75^NTR^, DR6, and the ectodomain of APP could assemble into a tripartite or even larger oligomeric complex to initiate downstream signaling pathways or undergo complex internalization in neuronal cells. Studies on the interactions between CRDs, transmembrane domains, and intracellular DDs of DR6 and p75^NTR^ are necessary to uncover the structural and functional details of DR6:p75^NTR^ complex in the presence of APP.

### Intracellular Signaling of DR6

DR6-mediated pro-apoptotic signaling does not require its intracellular domains to recruit the adaptor protein FADD and is independent of caspase 8 or Bid (BH3-domain-only protein). This suggests that DR6 is not involved in classical type I or type II pathways mediated by Fas, the first apoptosis signal death receptor. Instead, the apoptosis induced by DR6 strongly depends on the mitochondrial pathway when it is overexpressed (Figure 3A) (Zeng et al., 2012). Co-immunoprecipitation of DR6 and Bax (BCL2 associated X protein) indicates that DR6 may physically associate with Bax to transduce apoptotic signals. It is, however, unclear if this interaction requires an adaptor protein. Recently, Zhang et al. (2020) reported the interaction between DR6 and PSAP (PS1 associated protein) using a yeast two-hybrid assay (Zhang et al., 2020). PSAP is a transmembrane protein and localizes in the mitochondrial outer membrane, while DR6 is a plasma membrane receptor. It is less likely that DR6 could directly interact with PSAP due to their different subcellular localization unless DR6 could undergo internalization or enzymatic cleavage to release its intracellular domains. Similar to DR6, PSAP can also form a complex with Bax. Differently, DR6:Bax complex was only detected in the cytosolic fraction, while PSAP:Bax complex was only found in the mitochondrial fraction. Overexpressed PSAP can induce apoptosis, while knock-down of PSAP can prevent DR6-dependent Bax translocation and cytochrome C release in the mitochondria. On the other hand, knock-down of DR6 significantly reduces the formation of PSAP:Bax complexes in the mitochondria. It was therefore suggested that DR6-bound Bax could be transferred from DR6 to PSAP with an unknown delivery mechanism, resulting in mitochondria-dependent apoptosis.

In addition to the activation of the apoptotic pathway, DR6 was also shown to signal NF-κB upon overexpression in HEK293T cell lines (Figure 3A) (Hu et al., 2014). Interaction between DR6 and the adaptor protein TRADD was identified by co-immunoprecipitation. The DD of DR6 was responsible for TRADD recruitment, while the functional role of CARD domain remains elusive. It is very likely that TRADD relies on its death domain to interact with the DR6-DD. This interaction could enable TRADD to create a membrane-proximal platform in order to recruit downstream proteins to activate NF-κB signaling pathway, which is independent of the ectodomain of APP. The possibility that TRADD may also be involved in DR6-induced apoptosis is needed for further investigation.

## DR6-Related Disease

Currently, more and more emerging evidence has highlighted the pivotal role of DR6 signaling in the development of many human diseases, including tumor proliferation and metastasis, brain neurodegeneration causing Alzheimer’s disease, inflammation, and autoimmune disease. These findings may offer new therapeutic avenues for the treatment of DR6-related disorders.

### DR6 in Tumors

The expression level of DR6 is generally increased in various tumor tissues, including prostate, colorectal, lung, melanoma, gliomas, breast and ovarian cancers (Kasof et al., 2001; McNeal et al., 2016; Stegmann et al., 2019), suggesting an important role of DR6 in tumor development and progression. NF-κB activation induced by TNF-α and the expression of the anti-apoptotic protein Bcl-xL may promote DR6 expression and inhibit DR6-mediated apoptosis in tumor cells (TCs). The ectodomain of DR6 may also be specifically released by matrix metalloproteinase (MMP)-14 and form a soluble protein (sDR6) (Figure 3B). MMP-14 is highly expressed in tumor tissues, and MMP inhibitor GM6001 may significantly inhibit the production of sDR6 protein in the supernatant (Katsenelson et al., 2001; Tam et al., 2004; DeRosa et al., 2008). The free sDR6 not only affects the survival of differentiating monocytes but alters monocytes differentiation into immature dendritic cells (iDC) and thus prevent the development of anti-tumor DCs (DeRosa et al., 2008). It remains largely unknown how the ectodomains of DR6 interact with monocytes and immature DCs. Blocking the enzymatic cleavage of DR6 could provide an alternative way for promoting DC maturation (mDC) and survival and therefore boosting anti-tumor immunity.

DR6’s ligand, APP, is also abundantly expressed by TCs. DR6 on endothelial cells (ECs) can be activated by full-length APP from the surface of TCs through direct contact of ECs with TCs, leading to necroptosis of ECs and thus TCs extravasation and metastasis. The function of DR6 can be inhibited by an anti-DR6 antibody (5D10), which consequently protects ECs from TC-induced endothelial necroptosis and significantly weakens transendothelial migration of TCs (Strilic et al., 2016). Very recently, it was reported that a cell-permeable dimethyl-α-ketoglutarate can induce DR6 oxidation and endocytosis, leading to cell pyroptosis that may inhibit tumor growth and metastasis in mouse models (Zhang et al., 2021). These findings again suggest targeting DR6 has excellent potential for future anti-tumor therapies.

### DR6 in Neurological Disease

Axon degeneration is a common feature of a variety of nervous system disorders, and extensive nerve damage can trigger the process of axon self-destruction, leading to Alzheimer’s or other neurological diseases (Vohra et al., 2010). In pathological degeneration, neurons and branches are lost, leading to damage to neurons and ultimately axon degeneration. It was found that DR6 is highly expressed in the brain and nerve cells, and it can regulate the degeneration and death of neuronal cells and nerve axons (Wang et al., 2015). Hu et al. (2013) found that not only a large number of neuronal degenerations can be detected in samples of patients with Alzheimer’s disease, but the expression level of DR6 in the cerebral cortexes from the patients is significantly higher than that in the normal population (Hu et al., 2013). Blocking the function of DR6 or inhibiting the expression of DR6 can attenuate nerve cell apoptosis induced by exogenous Aβ stimulation (Huang et al., 2013).

In addition to Alzheimer’s disease, DR6 is also involved in the development of other neurological disorders. For example, DR6 is highly expressed in immature oligodendrocyte but negatively regulate maturation of oligodendrocyte through a mechanism independent of N-APP (Mi et al., 2011). Overexpressed DR6 in oligodendrocytes will lead to activation of caspase 3 and cell death. Treatment with DR6 siRNA and antagonist antibody can lower caspase 3 activation and significantly increase oligodendrocyte survival, maturation, and myelination. This result strongly suggests a critical role of DR6 in CNS (central nervous system) dysmyelination and demyelinating diseases. In the peripheral nervous system (PNS), the mechanism of DR6 action becomes different and more complicated. The ectodomain of DR6 can be cleaved by specific proteases, such as “a disintegrin and metalloprotease 10” (ADAM10), from the membrane and become soluble sDR6 (Figure 3B). sDR6 is then released from neurons and acts in trans on Schwann cells (SCs) to suppress their proliferation and thereby myelination, which is independent of DR6 cytoplasmic domains (Colombo et al., 2018). Therefore, deficiency of DR6 can result in precociousness of myelination in the PNS, a phenotype similar to that in the CNS.

### DR6 in Immune Diseases

DR6 is a crucial regulator of adaptive immunity. It is involved in regulating CD4^+^ T cell proliferation and helper T cell (Th) differentiation (Liu et al., 2001), as well as B cell expansion, survival, and humoral response, thus playing a critical role in immune diseases (Schmidt et al., 2003; Schmidt et al., 2005). After immunizing mice with myelin oligodendrocyte glycoprotein (MOG) peptide, mouse myelin-specific T cells can infiltrate the CNS, causing paralysis. However, DR6-deficient mice are highly resistant to the occurrence and development of this disease (Schmidt et al., 2005). Interestingly, the antigen recall response showed increased proliferation of CD4^+^ T cells in the isolated spleen of DR6-deficient mice. Transfer of myelin-specific T cells from DR6-deficient mice to wild-type mice did not cause the disease, but DR6-deficient mice that received myelin-specific wildtype T cells showed EAE (experimental autoimmune encephalomyelitis) symptoms (Schmidt et al., 2005). In another study, the induction of very late antigen 4 (VLA-4) was reduced when T cell from DR6-deficient mice was activated. Since VLA-4 is essential for T cell infiltration into CNS (Andreoli et al., 2007), DR6-deficient mice have an obvious resistance mechanism to induce EAE. Therefore, blocking DR6 function could be a potential strategy to regulate the migration of pathogenic T cells in patients with multiple sclerosis, an autoimmune inflammatory demyelinating disease characterized by an improper host immune response to its CNS antigens (Linker and Gold, 2021). Huang et al. (2013) demonstrated that the antagonistic antibody against DR6 has a neuroprotective effect in the SOD1G93A mouse model of amyotrophic lateral sclerosis (Huang et al., 2013).

DR6 also participates in regulating developments of lung eosinophilia and airway inflammation in mouse models of asthma, a highly reactive airway disease (Ohki et al., 2005). The immune response to asthma allergens is mainly triggered by a number of soluble factors and immune-mediated cells, including CD4^+^ T helper two cells, eosinophils, basophils, and neutrophils. These soluble factors include Th2 cytokines such as IL-4, IL-5, and IL-13 that play a role in inducing IgE production (Hahn et al., 2003). Compared with wild-type mice, DR6-deficient mice have reduced lung airway inflammation, which is evidenced by a decrease both in the number of eosinophils and Th2 cytokine production (IL-5 and IL-13) in bronchoalveolar lavage fluid (Venkataraman et al., 2006). This result is unexpected because previous studies showed that DR6-deficient mice have a hyperactive state and increased production of Th2 cytokine after being stimulated (Liu et al., 2001). The discrepancy could be due to the difference in immunization regimen and excitation method. Studies also suggested that cells lacking DR6 could be relatively defective in their ability to reach antigen sites (i.e., lungs). This hypothesis is supported by observations that T cells are activated in DR6-deficient mice of the EAE model while the cells do not infiltrate the area of antigen expression (Zhao et al., 2001). Therefore, future studies on the effect of DR6 on the expression of T cell adhesion molecules and chemokine receptors are necessary to reveal the mechanism of DR6 in asthma-related immune responses.

### Potential Drug Targets for DR6-Related Diseases

Since DR6 is closely related to a variety of human diseases, it has become a potential candidate for therapeutic approaches. Targeting the interactions between DR6 and its extracellular and intracellular interactors is feasible for developing drug candidates for the treatment of DR6-related diseases. These targets may include DR6:APP, DR6:TROY, DR6:p75^NTR^, DR6:TRADD, DR6:PSAP, and DR6:metalloproteinase complexes (Table 1). Very recently, a new type of conjugate inhibitor for the interaction between DR6 and APP, PEG-tAHP-DRI (tetra-(D-retro-inverso isomer of AHP-12) substituted 4-arm PEG) was synthesized as a promising agent to selectively block hematogenous TC extravasation due to the high binding affinity of this inhibitor to DR6 in the nanomolar range and low toxicity in mice (Wang et al., 2021). DR6 protein was also proposed to serve as a diagnostic or prognostic biomarker for some specific tumors, such as gliomas, adult sarcoma, and ovarian cancer since its level was obviously elevated in the serum or tumor tissues of the cancer patients (Sasaroli et al., 2011; Yang et al., 2012).

**TABLE 1:** Potential drug targets for DR6-related diseases.

Target	Drug candidate	Mechanism
DR6:APP	PEG-tAHP-DRI	Inhibition of DR6/APP interaction for protection against tumor cell extravasation (Wang et al., 2021)
DR6:TROY	N.A.	Promotion of DR6:TROY expression and interaction to treat vascular malformations and barrier defects (Tam et al., 2012)
DR6:p75^NTR^	N.A.	Blocking co-receptor formation for the treatment of Aβ-induced neuron degenerative disorders such as AD (Hu et al., 2013)
DR6:TRADD	N.A.	Blocking the first step of intracellular signaling for inhibiting NF-κB or apoptosis (Hu et al., 2014)
DR6:PSAP	N.A.	Blocking mitochondria-dependent apoptosis (Zhang et al., 2020)
DR6:metalloprotease	N.A.	Blocking tumor-derived DR6 cleavage for protecting DC differentiation and survival (DeRosa et al., 2008)

NA, not available.

## Conclusion and Outlook

During the last 10 years, advances have clearly increased our knowledge about DR6 as an orphan member of TNFRSF21. The ectodomain of DR6, probably together with its transmembrane domain, forms a homodimer on the membrane or a larger complex with its co-receptors, such as p75^NTR^ and TROY, to accept extracellular signals and initiate distinct downstream signaling, including unique mitochondria-dependent pathway. Although the free ectodomain of APP can bind to DR6 to induce the apoptotic signal, the natural ligands of DR6 still remain largely unknown. Current mechanistic studies are still far from deciphering the complete repertoire of DR6-mediated pathways leading to diverse cellular outputs. Nevertheless, limited structural information has already promoted rational structure-based drug design to develop potential candidates for the treatment of DR6-related diseases. Future identification of novel extracellular ligands, together with investigations on the unknown molecular and structural mechanisms underlying interactions between DR6 and its co-receptors, will provide clues and bases for targeting and manipulating DR6 therapeutically.
